# A programmable method for massively parallel targeted sequencing

**DOI:** 10.1093/nar/gku282

**Published:** 2014-04-29

**Authors:** Erik S. Hopmans, Georges Natsoulis, John M. Bell, Susan M. Grimes, Weiva Sieh, Hanlee P. Ji

**Affiliations:** 1 Stanford Genome Technology Center, Stanford University, Palo Alto, CA 94304, USA; 2 Division of Oncology, Department of Medicine, Stanford University School of Medicine, Stanford, CA 94305, USA; 3 Department of Health Research and Policy, Stanford University School of Medicine, 259 Campus Drive, Stanford, CA 94305, USA

## Abstract

We have developed a targeted resequencing approach referred to as Oligonucleotide-Selective Sequencing. In this study, we report a series of significant improvements and novel applications of this method whereby the surface of a sequencing flow cell is modified *in situ* to capture specific genomic regions of interest from a sample and then sequenced. These improvements include a fully automated targeted sequencing platform through the use of a standard Illumina cBot fluidics station. Targeting optimization increased the yield of total on-target sequencing data 2-fold compared to the previous iteration, while simultaneously increasing the percentage of reads that could be mapped to the human genome. The described assays cover up to 1421 genes with a total coverage of 5.5 Megabases (Mb). We demonstrate a 10-fold abundance uniformity of greater than 90% in 1 log distance from the median and a targeting rate of up to 95%. We also sequenced continuous genomic loci up to 1.5 Mb while simultaneously genotyping SNPs and genes. Variants with low minor allele fraction were sensitively detected at levels of 5%. Finally, we determined the exact breakpoint sequence of cancer rearrangements. Overall, this approach has high performance for selective sequencing of genome targets, configuration flexibility and variant calling accuracy.

## INTRODUCTION

With the widespread adoption of next-generation DNA sequencing (NGS), many human genomic studies utilize targeted approaches to identify variants from specific regions of the human genome. For example, targeted sequencing of all human exons has led to the discovery of cancer somatic mutations that are causative for oncogenic processes (1) and the identification of deleterious mutations leading to Mendelian disorders (2). Citing another application, many biological samples, such as tumor biopsies, consist of heterogeneous mixtures. Targeted sequencing with very high coverage is crucial in detecting less prevalent minor allele mutations and variants from these admixed samples.

Both academic and commercial groups have developed targeted sequencing approaches (1,3). These methods have multiple advantages including: (i) multiplexing large number of samples decreases the overall cost and analysis complexity of human genetic studies involving large populations (1,4,5); (ii) deep sequencing of specific genomic loci and higher read coverage improves variant calling accuracy, specifically in more complex genetically composed mixtures in which variants are present in lower allele frequencies (e.g. heterogeneous tumor samples) (6); (iii) targeting methods can be used to provide breakpoint resolution of complex structural variants such as rearrangements.

Most targeted sequencing methods require two discrete steps, an enrichment of the target followed by sequencing the target DNA. For the development of new applications and enrichment assays, this two-step process requires extensive optimization. Furthermore, most current targeting methods have a complex workflow and intricacies of preparation that make them prone to experimental error.

**Figure 1.**
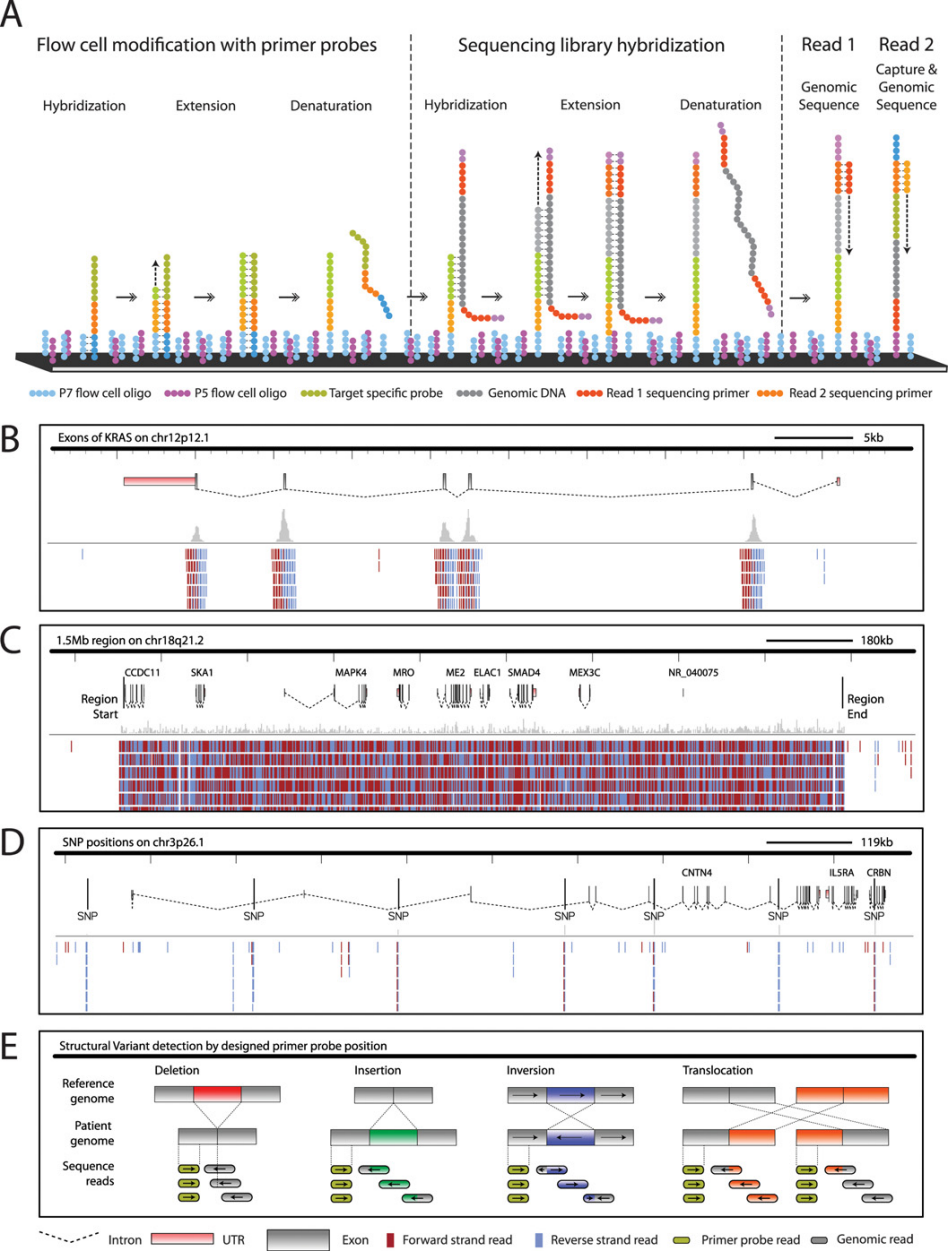
Programmable targeted sequencing: method and application. The following steps are required to program the sequencer for targeting genomic regions. (**A**) An Illumina flow cell is modified with strand and target-specific pools of primer probe oligonucleotides. These primer probes contain the P7 complementary region, which hybridizes randomly to the P7 primers of the flow cell primer lawn. Hybridized primer probes are covalently attached to the flow cell by a standard extension reaction and denatured to yield a target enrichment surface within the Illumina flow cell. Subsequently, a single-adapter sequencing library is introduced. The targeted library strands anneal to their complementary primer probes in an overnight hybridization. Primer probes are extended using the stringently captured library strands as template, followed by denaturation of the original library strands. The standard Illumina clustering reaction is performed to yield a ready-to-sequence flow cell. By tiling of primer probes, the target size can range from gene exons such as *KRAS* (**B**) to large genomic intervals such as a 1.5 Mb region on chromosome 18 (**C**) to individual SNP positions (**D**). Sequence target reads aligned to the human genome reference are depicted as per IGV. The primer probe is also sequenced in Read 2, which enables analysis of structural variants by comparing the genomic position and read direction of the sequence of genomic DNA to the primer probe sequence which has a known genomic position. This also enables assembly of breakpoints by grouping together sequence reads belonging to a unique primer probe sequence (**E**).

We developed Oligonucleotide-Selective Sequencing (OS-Seq), a flexible and efficient targeted sequencing approach for sequencing multiple genomic regions of interest (ROIs) (4). Unlike traditional bait hybridization strategies for target enrichment, this technology relies on hybridization of a genomic DNA library to a target-specific ‘primer probe’ located on the surface of an Illumina flow cell. Using the primer probe, a subsequent polymerase extension ‘selects’ the specific genomic target sequence. All steps of target selection occur on the same solid phase support that mediates the sequencing (Figure 1A).

This study details multiple improvements in the targeting process and development of a wide range of applications. In our initial study (4), we demonstrated the technical feasibility and performance metrics of OS-Seq on an Illumina Genome Analyzer*_IIx_* (GAIIx) with the earlier generation cluster station. In OS-Seq's original iteration, the critical processes of flow cell modification using the cluster station, including incorporation of primer probes, genomic library selection and flow cell preparation for sequencing, were an extensive hands-on process. In this study, we automate nearly all of these steps of OS-Seq flow cell preparation using the cBot fluidics station. The performance of the original method had some limitations. For example, our previous implementation using large primer probe pools (11 742 primer probe oligonucleotides) resulted in capture rates of only 47%. With advances in our primer probe design and experimental methodology, we demonstrate significant improvements in targeting performance that include more on-target sequence. With significantly expanded primer probe oligonucleotide pools, we show the feasibility of targeting over one thousand genes and Megabase (Mb) sized genomic loci.

In our original study, we demonstrated the performance on a limited number of assays. With these recent improvements, we can ‘program’ an Illumina NGS system for a diverse range of human genetic applications including the analysis of candidate gene sets (Figure 1B), variant detection in genetic mixtures, cancer mutation detection, Mb size contiguous loci from the human genome (Figure 1C), genotyping of specific Single-Nucleotide Polymorphisms (SNPs) (Figure 1D) and delineation of the sequence of structural variation breakpoints (Figure 1E). This final application involved developing a new sequence analysis method that relied on the sequencing from the targeting probe, as encoded in the second portion of a paired-end read, to precisely map out breakpoint sequences of structural variants.

## MATERIALS AND METHODS

### Genomic DNA samples

Genomic DNA from NA18507 was obtained from the Coriell Institute for Medical Research (Camden, NJ). Additional tissue and blood samples were obtained from the Stanford Cancer Institute Tissue Bank under a protocol approved by Stanford University Institutional Review Board. Genomic DNA from the blood and tumor samples was extracted using the E.Z.N.A. SQ DNA/RNA Protein Kit (Omega Bio-Tek, Norcross, GA). Concentrations of genomic DNA were determined with a Nanodrop instrument (Thermo Scientific, Wilmington, DE). Genomic DNA from blood, matched normal and cancer tissue were then used for creating sequencing libraries. From each sample, we fragmented 1 μg of genomic DNA with a Covaris E210R (Covaris, Woburn, MA) to a target base pair peak of 500 bp (settings: 5% duty cycle, intensity 3200 cycles per burst, 80 s). Fragmented DNA was purified using AMPure XP beads (Beckman Coulter, Brea, CA) in a bead solution to sample ratio of 1.0 X.

### DNA sequencing library preparation

The library preparation was modified from Myllykangas *et al.* (Nature Biotechnology, 2011) (4). Fragmented DNA was end-repaired at 25°C for 30 min using 5U of Klenow DNA polymerase, 50U T4 polynucleotide nuclease, 15U T4 DNA polymerase, 400 μM of each dNTP and 1× T4 ligase buffer w/ATP in a 100 μl reaction volume. Adenosine was added to 3′ ends of the repaired DNA strands at 37°C for 30 min using 15U Klenow Fragment (3′→5′ exo-), 200 μM of dATP and 1× NEB 2 buffer in a 50 μl reaction volume (all reagents from New England Biolabs, Ipswich, MA). Annealed duplex adapters were ligated to the A-tailed DNA at 25°C for 1 h in a final concentration of 0.15 μM with 2000U T4 DNA ligase and 1× T4 ligase buffer w/ATP in a 25 μl volume. Every step of library preparation was followed by AMPure XP bead purification in a sample to bead ratio of 1.0 X. We prepared 50 μl reactions for library amplification using polymerase chain reaction (PCR), containing 25% of adapter annealing step product, 1 μM amplification primer and 25 μl KapaHiFi Hot Start Mastermix (KapaBiosystems, Woburn, MA). Reactions were denatured at 98°C for 30 s, followed by 14 cycles of 10 s of 98°C, 30 s of 65°C and 30 s of 72°C. The final steps involved an incubation at 72°C for 7 min and cooling to 4°C. Amplified libraries were purified with AMPure XP beads in a bead solution to sample ratio of 1.0, and a Nanodrop spectrophotometer (Thermo Scientific, Wilmington, DE) was used for quantitation. The expected library length of 700 bp was confirmed by gel electrophoresis.

### Primer probe design for exons, SNPs and contiguous genomic loci

To facilitate the programmable design of primer probes, we identified specific sequence parameters to facilitate the targeting of multiple regions throughout the human genome. This was conducted with a series of Matlab scripts (Mathworks, Natick, MA). We developed a pre-computed method for selecting primer probe sequences. Using a single base offset, we generated all of the 20-mer sequences from the 37.1 (hg19) release of the human genome. We determined the mapability of these 20-mers in terms of their unique or repetitive location in the human genome, taking into account exact matches or tolerance for one to two mismatches.

Our bioinformatic pipeline optimizes the placement of primer probes based on several criteria including (i) placing primer probes upstream and downstream of any given target for double-stranded coverage of the targeted region within 100 to 200 bases; (ii) adjustable primer probe density; (iii) a primer probe GC content between 30% and 65%; (iv) uniqueness of the last 20 bases for the 3′ portion of the primer probe in the human genome with a string edit distance of 1 from any other genome location; (v) no overlap of the last 10 bases with a known SNP as annotated in dbSNP Build ID 131; (vi) no sequences immediately adjacent to highly repetitive sequences. The 20-mer data files and Matlab scripts are available for download from http://dna-discovery.stanford.edu/software/osseq/.

### Primer probes and oligonucleotides

The top (5′-CGAGATCTACACTCTTTCCCTACACGACGCTCTTCCGATC*T), which contains a phosphorothioate bond (indicated by *), and bottom (5′-/5Phos/GATCGGAAGAGCGTCGTGTAGGGAAAGAGTGTAGATCTCG) singleplex adapters were HPLC-purified (IDT, Coralville IA). For the multiplex adapters, which contain a 7-base indexing sequence (xxxxxx*T) directly following the sequencing primer binding site (top: 5′-CGAGATCTACACTCTTTCCCTACACGACGCTCTTCCGATCxxxxxx*T; bottom: 5′-/5Phos/xxxxxxGATCGGAAGAGCGTCGTGTAGGGAAAGAGTGTAGATCTCG), we used standard desalted ultramer oligonucleotides (Integrated DNA Technologies, Coralville, IA).

Both singleplex and multiplex adapters were annealed in a final concentration of 15 μM per adapter in Nuclease Free Duplex Buffer (IDT) by a 1% temperature ramp from 94°C to 20°C, after an initial 5 min 94°C denaturation step. Unlike standard Illumina adapters, our modified library adapters are only complementary to the P5 primer on the flow cell surface (Supplementary Table S1). The portion that is complementary to the P7 primer is introduced in the primer probe extension step (Figure 1A). For Assays 1 and 5, primer probes were column-synthesized at the Stanford Genome Technology Center and combined to an isomolar pool. For Assays 2, 3 and 4, we purchased array-synthesized oligonucleotides that had been amplified and purified to single-stranded DNA form by the vendor (Mycroarray, Ann Arbor, MI).

### Programmable sequencing of genomic target regions

To program the flow cell for targeting, we generated a modified XML script for the Illumina cBot (Supplementary Table S2) (Illumina, San Diego, CA). The modification process requires (i) hybridization and extension of the target oligonucleotides onto the flow cell primer lawn and captures the sequencing library by overnight hybridization; (ii) extends the captured library and performs standard Illumina cluster generation. We developed XML scripts specific to the Illumina GAIIx or HiSeq systems.

Oligonucleotides and the sequencing library were heat denatured for 5 min at 95°C and directly followed by incubation on ice. Afterward, we diluted both components with ice-cold 4× hybridization buffer (20× SSC, 0.2% Tween-20) to a final total concentration of 75–100 nM for the primer probes and 50 ng/μl for the sequencing library. Denatured primer probes and libraries (both 50 μl) were loaded in separate eight tube strips. We created a custom cBot reagent plate (Supplementary Table S3), containing hybridization buffer 1 (pos.1: HT1 or 5× SSC, 0.05% Tween-20), extension mix (pos.2: 20U/ml Phusion (Thermo Scientific); 0.2 mM dNTP; 1x Phusion HF buffer), pre-extension mix (pos.3: 1× Phusion HF buffer; used with GAIIx only), wash buffer (pos.7: HT2 or 10 mM Tris buffer) and freshly prepared 0.1 N NaOH (pos.10).

The reagent plate and eight tube strips containing the denatured primer probes were loaded onto the Illumina cBot. We set the ‘Wash before Run’ and ‘Wash after Run’ setting (i.e. Menu/Configure) to Optional. In the RunConfig.xml file, we increased the number of cycles to 42 (i.e. Amplification MaxNumCycles). Two different cBot programs were used for the subsequent steps. The first cBot program (P1) automates the hybridization and extension of the primer probes to a subset of the P7 primers of the flow cell surface, followed by denaturation and removal of the original primer probe oligonucleotides. Finally, the denatured sequencing library is hybridized to the generated primer probe capture flow cell lawn in an overnight hybridization at 65°C.

After the completion of the P1 program, the second cBot program (P2) is started. This program performs a stringency wash of the hybridized library, followed by the standard Illumina extension and clustering protocol. The standard Illumina cBot clustering reagent plate is used for this process. For runs performed on the Illumina GAIIx we generated either 60 by 60 or 80 by 40 paired-end cycles using cBot clustering reagents v2 and sequencing reagents v5 (Illumina). For the GAIIx, image analysis and base calling were performed using the SCS 2.9 and RTA 1.9.35 software (Illumina). For runs performed on the Illumina HiSeq, we used cBot clustering reagents v3 and sequencing reagents v3 (Illumina) for either 60 or 100 cycle runs. For the HiSeq, image analysis and base calling were performed using the HCS 1.5.15.1 and RTA 1.13.48 software (Illumina).

### Targeted sequence analysis

For multiple sample indexing, we generated an index of the 7-base tags using the base-call file to assign reads to the correct sample (1). With default settings, we used Burrows-Wheeler Aligner (BWA) (7) to align sequences to the human genome version human genome build NCBI 37 (hg19). We relied on Samtools (8) for additional sequence processing and coverage analysis. Since primer probes have shown to occasionally capture sequences over a 1 kb from the primer probe loci, we called reads off-target when they aligned with an insert size larger than 1.5 kb between sequence read and primer probe.

We determined on-target coverage numbers with the program Bedtools (9) using the coverageBed command. For each assay, we created a series of bed files for target regions; this involved using the location of the primer probes and then enlarging the interval by 50 bases on each flank. To eliminate synthetic sequences from the primer probe, we only used sequence reads that did not overlap with the primer probe sequence. Samtools was used to create mpileup files (settings -B -d100000000 –q 15). VarScan 2.3.3 (10) was used for variant calling (settings --min-coverage 10 --min-var-freq 0.15 --min-avg-qual 25 --p-value 0.05 for mpileup2snp with addition of --somatic-p-value 0.05 for somatic analysis of tumor normal samples). We used the Integrated Genome Viewer (IGV) (11) to visually inspect sequence reads and variant positions. From the analysis of the normal tumor pairs, somatic mutations leading to amino acid substitutions were assessed using three different prediction algorithms: Provean (12), SIFT (13) and PolyPhen2 (14). Mutations were considered pathogenic candidates if they were called in all three algorithms (cut-off values: Provean < −2.5; SIFT: <0.05; PolyPhen2: >0.95).

For structural variation analysis performed, we used reads from the putative breakpoints that had a Phred-like score greater than 25. We aggregated the sequence reads for each rearrangement loci, generally referred to as locus groups. Subsequently, for each locus group, sequence reads that matched the reference genome were eliminated, as were reads containing sequencing primer 2 (AGATCGGAAGAGCGGT or its reverse complement ACCGCTCTTCCGATCT) to prevent contig formation on these sequences. Based on locus grouping, the remaining non-aligning reads were subject to localized assembly using Velvet (15) with the remaining reads. Parameters for Velvet included a hash length of 19, a contig length-minimum of 50 and a contig coverage depth minimum of 4. The generated contig sequences were aligned with megablast (16) against the human genome reference and considered based on their location on the correct chromosome, discontinuous sequence alignment starting from the breakpoint and appearance only in the cancer genome compared to the matched normal genomic DNA.

### Whole genome sequencing analysis

Whole genome sequencing libraries were clonally amplified through cluster generation on an Illumina cBot using paired-end flow cells and Illumina TruSeq v2 chemistry. Using PicoGreen assay (Invitrogen, San Diego, CA) for quantitating the amount of library, we prepared samples for input according to the Illumina cBot User Guide. The clustered flow cell was sequenced on an Illumina HiSeq 2000 for 2 × 100 cycle reads with indexing, using Illumina TruSeq v2 reagents. Sequence reads were aligned using BWA.

Data were analyzed using Breakdancer with breakdancer_max. To be considered as a potential variant, we required an anchor sequence of 20 base pairs on each side of a rearrangement breakpoint (breakdancer_max -t -s 20 -r 10 configfile). We also filtered out implausible cases (e.g. involving Y in female), required a minimum number of reads (20 for an individual genome finding or 10 common to two different genomes) and eliminated calls seen in the normal germline genome. For cancer-specific, intra-chromosomal events such as large genomic deletions, we required at least 20 reads to cover the putative breakpoint of the event which were seen in the primary or metastatic cancer but not the normal germline genome. As a final filter, we eliminated putative structural variants where the anchor sequence occurred in highly repetitive sequences that were a potential source of mapping errors.

## RESULTS

### General description of programmable target sequencing

For this study, we employed the Illumina cBot fluidics station for mediating selection of genomic ROIs and preparation of the flow cell for sequencing. We reprogrammed the cBot to handle all of the temperature ramping and enzymatic reactions, thus streamlining the overall process and minimizing the hands-on preparative time. For example, a ready-to-sequence flow cell is generated in ∼27 h with the only requirement being the straightforward preparation of a genomic DNA sequencing library and loading of reagents. There is no requirement for library size selection given that we rely on a size range produced by the fragmentation process. Sequencing was performed on an Illumina GAIIx or HiSeq system.

To modify or program the primer lawn on the inside surface of the Illumina flow cell into a target enrichment surface, the initial step is designing 101-mer ‘primer probe’ oligonucleotides (Figure 1A) with the following components: (i) a 5′ target-specific 40-mer sequence flanking a genome-wide ROI; (ii) a universal sequencing primer; (iii) a universal sequence complementary to the existing lawn of P7 primers fixed to the surface of the flow cell.

Empirical data obtained during development of the original assay led to improvements in the design process of the 5′ target-specific 40-mer sequence (see the ‘Primer probe design and synthesis’ section). Primer probe oligonucleotides do not contain modified nucleotides and were generated via column or microarray-based synthesis. For this study, we have created pools comprising up to 90 000 unique primer probe oligonucleotides. As discussed later, expansion of targeted regions can be accomplished by simply combining pools of primer probe oligonucleotides.

Using an adapted protocol on the Illumina cBot fluidics system, these primer probe pools are used to modify and thus ‘program’ the already present primer lawn on the inside of an Illumina flow cell. The 3′ P7 universal complementary region of the free primer probe oligonucleotide hybridizes to the P7 primer lawn of the flow cell. Next, we incorporate the target-specific primer probe onto the flow cell with a polymerase extension reaction, denature and then remove the original primer probe oligonucleotide (Figure 1A). Ultimately, this process results in a lawn of single-stranded target-specific primer probes that are immobilized to the flow cell surface. As a result, the flow cell becomes a target-specific selection and enrichment device.

Afterward, we hybridize a DNA sequencing library against the target-specific regions of the fixed primer probes. The sequencing libraries are generated by randomly shearing genomic DNA to an average size of 500 bases, followed by end-repair, A-tailing and single-adapter ligation. The target selection process tolerates the fragmentation size range, which removes the need for sequencing library size selection. The adapter only contains part of the P5 primer complementary sequence, but not the P7 primer of the flow cell lawn as the original primer probe already introduced this sequence. As a result of this adapter design, hybridization of the library against the target-specific primer probe 40-mer is favored rather than the adapter against the non-modified flow cell lawn. An overnight hybridization reaction of the denatured library to the targeting prime surface occurs for 20 h.

The yield of hybridization is time dependent (Supplementary Figure S1) and library concentration dependent. We calculated previously that after 20 h of hybridization with 500 ng of sequencing library, ∼4.9% of all potential targets within the sequencing library were captured for sequencing (4). Therefore, library fragments are available in excess for optimal capture and do not require exact titration. We did observe a large drop in sequence yield when half the concentration of sequencing library was used; an increase in the library concentration did not lead to a significant increase in on-target sequence.

Following target hybridization, the primer probe provides a start site for polymerase extension of the captured library, which incorporates the target sequencing library strand to the primer probe fixed on the flow cell surface (Figure 1A). An important aspect of OS-Seq's capture-extension approach is that each individual sequencing library DNA molecule is directly incorporated onto the flow cell and thus generates a unique sequencing cluster. To some degree, this reduces the post-capture PCR bottlenecking as seen with other methods.

The last step of the flow cell modification involves a DNA polymerase extension reaction to complete the portion of the adapter (e.g. P5 sequence) (Figure 1A). The selected genomic regions undergo the standard Illumina bridge-PCR clustering reaction and sequencing primer hybridization, completing the flow cell's preparation for sequencing.

In the case of paired-end sequencing, the first read (Read 1) comes from the selected genomic target, while the second read (Read 2) covers the synthetic target-specific primer probes and adjacent genomic target sequence. As mentioned, during the initial development we used a first-generation Illumina cluster station (4) for flow cell preparation. Our current cBot-based protocol increased the total number of reads over 2-fold compared to the older cluster station system and increased the percentage of aligning reads compared to the initial study (Supplementary Figure S2).

### Primer probe design and synthesis

We developed a process for identifying optimal primer probe sequences for any arbitrary target in the human genome. From our previous studies (4), we determined that the final 20 bases at the 5′ end target-specific 40-mer sequence (the 3′ portion after primer probe immobilization on the flow cell) are the most critical component for target specificity and efficient target selection by polymerase extension.

As the initial computational step, we aligned all 20-mer sequences in the human genome (NCBI 37.1/hg19) *in silico* and determined the uniqueness of each 20-mer sequence. Candidate primer probes were restricted to the 20-mers that were unique in the human genome and required at least two edited bases to align elsewhere in the genome. The remaining 20 bases were then added; this step completes the target-specific 40-mer sequence of the primer probe. Other design parameters include: (i) incorporating double-stranded coverage of the targeted genomic region by selection of primer probes in both forward and reverse strand orientation; (ii) attempting to place primer probes approximately every 200 bases on each strand; (iii) primer probe GC content between 30% and 65%; and (iv) no known SNPs (as annotated in dbSNP31) in the last 10 bases on the 5′ side of the primer probe, since this is the region most crucial for successful hybridization. The primer probe design pipeline is available for public download on http://dna-discovery.stanford.edu/software/osseq/. We also have provided our primer probe sequences for all of the assays in the supplementary section.

Flanking a genomic target with multiple primer probes on opposing strands enables sequencing of both the forward and reverse strands in a genomic target interval. As we previously demonstrated, a true variant is typically seen in reads from both the forward and reverse strands (17). This feature of tiling the forward and reverse strands in targeting primer probes enabled very dense distribution of primer probes to target extended genomic intervals larger than exons.

For massively multiplexed gene sequencing, we relied on the exon definitions provided by the Consensus Coding Sequence Project (Release 9, 7 September 2011) (Figure 1B). Subsequently, we chose lists of genes related to disease or biological processes and generated sets of related oligonucleotide sequences covering exons and 50 bases of adjacent intronic sequence and selected appropriate primer probes covering these targets (Table 1). Exons in the range of 1 kb or larger relied on a tiling strategy every 200 bases. The average primer probe density was 7.85 probes per 1 kb. A dense distribution of forward and reverse strand oriented primer probes cross the entire length of the exon.

**Table 1.** Description of targeting assays

Multi-target assay	Application	Targeted genes	Targeted exons^b^	Other regions of interest^b^		SNPs^b^	Targeted region (Mb)	Number of primer probes	Oligonucleotide synthesis method
Assay 1	RAS/RAF/MAPK pathway genes	29	517				0.127	1943	Column-synthesis
Assay 2	Cancer gene set^a^	313	5592				1.470	19532	Microarray
Assay 3	Cancer gene set^a^	1421	21 069				5.500	90000	Microarray
Assay 4	Contiguous loci and SNP genotyping	47	999	1.5 Mb coverage of chr 18q21.1	0.2 Mb region spanning *TIPARP* on Chr 3	1701 SNPs - Chr 3	2.070	17548	Microarray
Assay 5	Cancer rearrangement detection	29	517	66 cancer rearrangements	Genes from Assay 1		0.175	2912	Column-synthesis

^a^Ranked highly for cancer involvement in Generanker database by Gonzalez *et al.*(18).

^b^Region of interest is defined as a minimum of the exon or non-exonic target and adjacent sequence up to 50 bases from the target flank.

Several assays were designed for covering all of the exons for specific disease and cancer genes, and all of the primer probe sequences are provided in the Supplementary Data. Assay 1 includes 29 genes, covering a combined target region of 0.127 Mb (Supplementary Tables S4 and S5). This assay includes genes involved in mediating Ras/Raf/MAPK signal transduction, an important oncogenic pathway in cancer. Assays 2 and 3 cover the exons of 313 and 1421 genes, respectively (Supplementary Tables S6, S7 and S8); the majority of these genes are ranked highly by GeneRanker (18) for cancer involvement.

Primer probe sequences can be tiled and thus cover a range of genomic interval sizes from the local sequence around a SNP to as large as extended contiguous loci in the order of 1 Mb or greater (Figure 1B, C and D). Using our pipeline, we designed several targeting assays that had coverage of non-coding regions (Table 1). Assay 4 was designed to target both contiguous large target regions and individual SNPs. Two contiguous target regions were included: a 0.2 Mb interval, the span of the *TIPARP* gene and flanking regions (Chr 3: 156299721–156500330), and a 1.5 Mb region covering a portion of chromosome region 18q21.1 (Chr 18: 47749745–49250310) that is frequently subject to deletion in colon cancer (19). Assay 4 also targets 1701 individual SNP positions on the flanking regions of the *TIPARP* locus and the exons of 46 additional genes involved in breast cancer (Supplementary Tables S4 and S8). Primer probes for Assay 5 were designed to target novel breakpoint sequences arising from rearrangements in a gastric cancer. We used this assay to obtain high coverage sequence data for validation of candidate structural variant regions and identification of the exact breakpoints by local assembly. Assay 5 also covers gene exons from Assay 1 by pooling together oligonucleotides (Supplementary Tables S4, S5 and S9).

We used two different methods of oligonucleotide synthesis (Table 1) in designing assays. For the smaller primer probe sets (Assays 1 and 5), we used traditional column-synthesized oligonucleotides. For the larger gene sets (Assays 2, 3 and 4), primer probes were synthesized on a programmable microarray, since array-synthesized oligonucleotides allow generating tens of thousands of oligonucleotides rapidly and cost effectively, although they require the additional step of amplification and purification.

### Characteristics of on-target sequence coverage from assays targeting genes

Reads aligning within 1 kb of a primer probe and in the expected orientation were considered to be on target. The average percentage of on-target sequence was 86.0% for Assay 2 (19 532 primer probes), 93.0% for Assay 3 (90 000 primer probes) and 74.8% for Assay 4 (17 548 primer probes). These percentages were reproducible in different experiments using the same assay pool (Table 2).

**Table 2.** Sequencing metrics and variant calling summary

Sample	Primer probe pool	No. of total reads	Mapped reads (% of total reads)	On-target reads^a^ (% of mapped reads)	Average coverage on ROI^b^	Percentage of ROI^b^ with at least 1×/10×/30× coverage	SNV calls from ROI^b,c^	SNV calls reported in dbSNP137^c,d^ (%)	SNV concordance to validation data set
NA18507	Assay 1	50549953	48481205 (95.9%)	36569769 (75.4%)	7017	100% / 99.6% / 99.4%	96	91 (94.8%)	98.9%
2546	Assay 2	19499716	15827573 (81.2%)	12813301 (81.0%)	358	99.5% / 98.4% / 96.0%	977	965 (98.8%)	96.6%
2546	Assay 3	10690321	8683523 (81.2%)	8078055 (93.0%)	67	97.4% / 85.4% / 66.9%	3311	3239 (97.8%)	97.4%
NA18507	Combined Assays 1 and 2	20281694	13821438 (68.1%)	11352137 (82.1%)	440	99.6% / 98.8% / 96.5%	1205	1184 (98.3%)	96.0%
168 Tumor	Assay 2	8289245	7038272 (84.9%)	6234236 (88.6%)	160	99.5% / 97.7% / 92.2%	1025	940 (91.7%)	-
168 Normal	Assay 2	8111210	7008081 (86.4%)	6200732 (88.5%)	155	99.4% / 96.8% / 89.7%	961	904 (94.1%)	-
5614	Assay 4	4346138	3744698 (86.2%)	2791668 (74.5%)	74	97.9% / 90.2% / 73.0%	2497	2467 (98.8%)	98.0%^e^
6235	Assay 4	4943719	4235356 (85.7%)	3139158 (74.1%)	84	98.1% / 91.9% / 76.5%	2515	2486 (98.8%)	99.2%^e^
5326	Assay 4	4804386	4126371 (85.9%)	3083111 (74.7%)	80	98.1% / 91.4% / 75.7%	2566	2528 (98.5%)	98.4%^e^
5613	Assay 4	4580779	3935242 (85.9%)	2986708 (75.9%)	82	97.8% / 90.3% / 74.0%	2484	2447 (98.5%)	97.9%^e^

^a^Reads within 1000b from the primer probe are considered to be on target.

^b^Region-of-interest is defined as the exon or non-exonic target region and adjacent sequence up to 50 bases from the target flank.

^c^Mapped within 1000 bp from primer probe, filtered insert size ≥ 40+ Read 1 length.

^d^http://snp.gs.washington.edu/SeattleSeqAnnotation137/index.jsp.

^e^Illumina BeadChip genotyping data.

### Characteristics of improved primer probe design

When generating paired-end reads, the first 40 bases of Read 2 constitute the primer probe synthetic sequence (Figure 1A), followed by genomic sequence from the targeted region. The synthetic sequence from the primer probe serves as a target-specific index for any given paired-end read set. We use this feature for delineating the performance of individual primer probes.

To measure the targeting performance of Assays 2, 3 and 4, we determined the number of reads associated with a specific synthetic primer probe sequence. All pools in this study were created with equimolar oligonucleotide concentrations; we did not rebalance concentrations to improve capture performance of primer probes with lower yields. Generally, one sample was sequenced on a single lane of an Illumina GAIIx or HiSeq 2000 with the exception of multiplexed samples that are otherwise described. We used the primer probe sequence obtained in Read 2 as an index for grouping the appropriately paired Read 1. We observed in our original assay (4) that ∼47% of the 11 742 primer probes captured target sequence. In contrast, Assays 2, 3 and 4 had 98.1%, 92.6% and 95.5%, respectively, of the primer probes with on-target sequence. When compared to the original assay using 11 742 primer probes, Assays 2, 3 and 4 were 1.5, 7.7 and 1.6 times larger, respectively. Therefore, we achieved higher on-target performance with significantly larger primer probe pools compared to our first effort.

**Figure 2.**
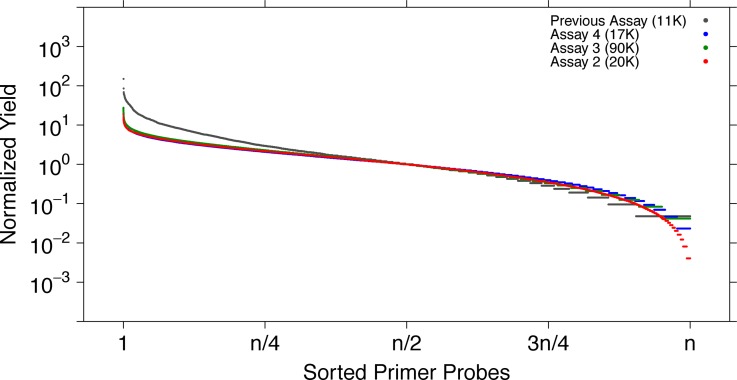
Improved uniformity of genomic target selection. Optimized Kmer-based primer probe design with maximum target specificity improves selection uniformity of newly designed microarray-produced pools. We analyzed all 20 base Kmers in the genome (NCBI 37.1) for unique alignment position (and required at least two edited bases to align elsewhere in the genome), which has enabled optimal placement of primer probes with adjustable densities and optimal primer probe criteria, such as 30–65% GC content, exclusion of repeats, up and downstream of selected target and no overlap with known SNPs (dbSNP131) in the last 10 bases. We compared the uniformity of capture of the array-synthesized pools between previous design (gray, 11 742 primer probes) and new design (with generated primer probe pools (Assay 2, red, 19 532 primer probes; Assay 3, green, 90 000 primer probes; Assay 4, blue, 17 548 primer probes)). Yield uniformity improved greatly with the new design, while increasing the number of unique primer probes 1.7- to 7.7-fold. Primer probes are sorted on their yield on the x-axis and the normalized -median-based- primer probe yield is depicted on the y-axis.

We determined the target-specific yield that occurred within one order of magnitude from the median across all of the primer probes to be 90.1%, 90.4% and 91.6% for Assay 2, 3 and 4, respectively. Both improvements were also observed using the original cluster station and thus can be attributed to improved primer probe design. Overall, these results demonstrated a high capture efficiency and uniformity among the different pools; this was a significant improvement over our previous efforts (Figure 2). The assays showed this level of performance without rebalancing of individual primer probe concentrations.

### Targeted genomic sequencing and germline variant calling

To determine variant calling performance, we sequenced normal diploid genomes that had been previously subject to exome sequencing and compared our variant calling results to the previous findings derived from the exome data. Sequence data were aligned with BWA (7) and variants were called using VarScan2.3.3 (10) (see the Materials and Methods section). Afterward, variants were annotated with the SeattleSeq web site (20).

With Assay 1, we sequenced the normal DNA of a Yoruban individual (NA18507) who was included in the Hapmap and 1000 Genome Projects. This involved a single lane of a HiSeq. This individual has already been extensively whole genome and exome sequenced (21). The variant calls from high coverage exome analysis (22) were compared to our results. Assay 1 covers 29 genes that have a total of 517 exons (Tables 1 and 2). Overall, 74.5% of the sequence reads were on-target (defined as within 1 kb of the primer probe), with an overall average sequencing coverage of 7017×. In the target ROI, over 99% of the bases had sequencing coverage greater than 30×. We identified 89 single nucleotide variants (SNVs), of which 88 were concordant with the exome-based variants (Table 2, Supplementary Table S10). The single discordant variant was apparent in the sequence data from Assay 1, but the variant caller (e.g. Varscan) eliminated these reads as a result of a low mapping quality less than 15.

To determine the performance of the larger exon gene panels of Assays 2 and 3 (Table 1), we sequenced an individual (e.g. 2546) previously analyzed with three different exome capture methods (23) and compared our results to the exome germline variants. We used the previously reported variants that were concordant in at least two of the exome capture methods. Assay 2 covered a total of 313 genes and all of their exons (Supplementary Table S4). We used a single lane of a GAIIx. For this analysis, we generated 19.5E6 reads with 81.0% of the mapped reads being on-target for the selected genomic regions. The average fold coverage on the targeted regions was 358×. At least 96% of the targeted bases had greater than 30× coverage.

From this data, we identified 977 SNVs, of which 98.8% were previously annotated by dbSNP137 (Supplementary Table S11). For the target region, the concordance of our SNVs compared to the exome analysis SNVs was 96.6%. Our approach detected an additional 10 variants of which three were novel and seven were detected by at least one of the three exome capture methods in our reference set. The majority of non-concordant variants were derived from two target genes, *MLL3* and *FANCD2*, that have shared sequence motifs with other gene families; this phenomenon likely increased the off-target yield. For example, the *MLL3* gene is duplicated on chromosomes 9, 13, 18 and 21 as a result of the juxtacentromeric reshuffling of the *BAGE* gene family (24). We observed reads aligning to the *BAGE* genes that paired with primer probes targeting the *MLL3* gene, diluting the variant coverage to an undetectable level. In addition, we observed many reads aligning to the *FANCD2* gene with very low mapping quality. These reads were excluded from variant detection. Four of the remaining non-concordant variants were not called by Varscan because of a low *p*-value but were present in the sequence on visual inspection.

Assay 3 covers the exons of 1421 genes, the majority of which play a role in cancer development and maintenance as denoted by GeneRanker (Table 1). Using a single sequencing lane on a GAIIx, when Assay 3 was used to sequence Individual 2546's genomic DNA, the overall sequence yield was 10.7E6 reads with 93.0% being on target for the selected genomic regions. For the targeted regions, the average fold coverage was 67×. At least 66.9% of the targeted exons had greater than 30× coverage. From this data, we identified 3311 SNVs, of which 97.8% were previously annotated by dbSNP137 (Supplementary Table S12). The concordance of our SNVs compared to the previously reported variants was 97.4%. Similar to Assay 2, half of the non-concordant variants align to the *MLL3* and *FANCD2* genes, with addition of the *PDE4DIP* gene. *PDE4DIP* has a paralogous region in the p-arm of chromosome 1, which given the duplicated structure is a likely source of false positive calls.

To demonstrate the ease of assay expansion, we combined Assay 1 and 2 into a single pool of free oligonucleotides. Subsequently, we sequenced the NA18507 DNA sample with the enlarged primer probe set. For this combined assay, we generated 20.2E6 reads from a single lane of a HiSeq 2000 with 68.1% of the mapped reads being on-target for the selected genomic regions. The average fold coverage on the targeted regions was 440×. At least 96% of the targeted bases had greater than 30× coverage. Regarding variant calling quality, among the 1205 SNVs called, 98.3% were previously annotated in dbSNP and 96% were concordant with SNVs from a previous exome analysis (Supplementary Table S13).

### Identifying minor allelic variants in a genetic mixture

We tested the sensitivity of our targeting approach in detecting variants with minor allelic fraction (MAF). This experiment involved a series of genetic mixtures with varying ratios of two samples. We used normal diploid genomic DNA from individuals 525 and 2546, both of whom had been previously subject to extensive whole genome and exome sequencing.

**Figure 3.**
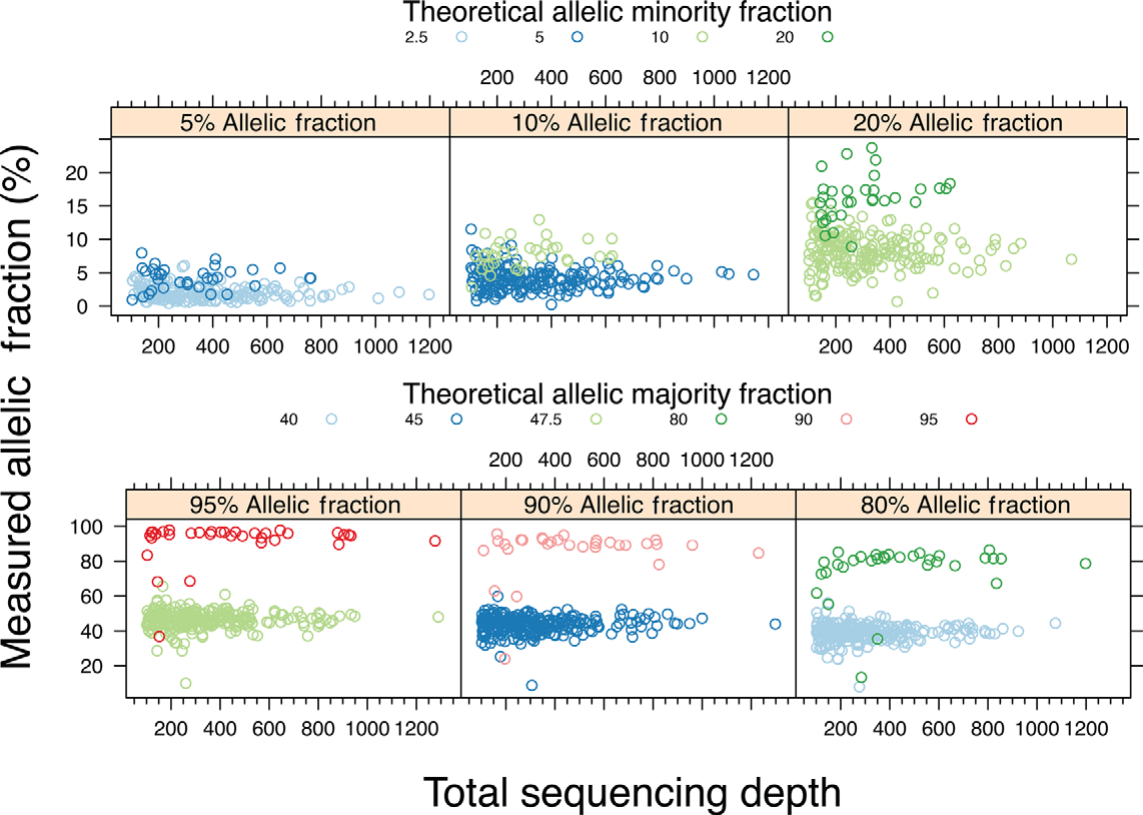
Low allele fraction detection. Allelic fractions of low abundance variants are proportionally detected. To determine our capability to detect low variant frequencies, we mixed two DNA samples in different ratios (5%, 10% and 20%). Subsequently, we generated sequencing libraries that were analyzed with Assay 2. A variant subset was unique for either genome in the target region. These variants were grouped by theoretical allelic fraction for each genome (homo- or heterozygous). The allelic fraction of the variant was plotted against the total sequencing depth. Both minor allele (left to right: 5%, 10% and 20%) (**A**) and majority allele (**B**) samples detect variants proportionally, with reduced noise at higher sequencing depth.

Individual 2546 had been analyzed with Assay 2 as described previously. Genomic DNA was combined in 5%, 10% and 20% weight ratios of individual 525's DNA spiked into individual 2546's DNA (Table 3). A total of 644 positions were unique for one individual or the other; 294 positions were specific to individual 525. Relying on targeted regions with a sequencing coverage greater than 100×, we determined whether we could identify the variants unique to 525. In the 20% spike-in data, there are 224 variant positions unique to individual 525; 223 were detected for a variant detection rate of 99.6%. For the 10% and 5% spike-in, there are 232 and 240 variant positions, which lead to detection rates of 99.6% and 93.3%, respectively. As demonstrated in Figure 3, high sequencing depth is required for exact proportional detection of low abundance variants.

**Table 3.** Variant calling from genetic mixtures

	Heterozygous variants		Homozygous variants		Total variants	
	Nr of variants	Detected (%)	Nr of variants	Detected (%)	Nr of variants	Detected (%)
Total unique comparable variants^a^	248		46		294	
20% spike^b^	192	191 (99.6%)	32	32 (100%)	224	223 (99.6%)
10% spike^b^	198	197 (99.5%)	34	34 (100%)	232	231 (99.6%)
5% spike^b^	202	191 (94.6%)	38	33 (86.8%)	240	224 (93.3%)

Number (Nr).

^a^Comparable variant positions unique for individual 525.

^b^Compared positions with a minimal sequencing depth of 100.

### Cancer mutation discovery

As noted previously, Assay 2 targets 313 cancer genes and this assay was employed in the analysis of a matched colorectal tumor-normal tissue pair (individual 168). Sample indexing allowed the normal and tumor pair to be run in a single sequencing lane (see the Materials and Methods section). We generated 8.29E6 and 8.11E6 total reads for the tumor and matched normal tissue, respectively, from a single sequencing lane. The tumor data had 88.6% on-target reads and 160× average coverage, while the matched normal tissue had 88.5% on-target reads and 155× average coverage.

Somatic variant calling was conducted with Varscan2 on the matched sample sequence data (Supplementary Table S14). We identified 94 somatic mutations that occurred in exons; these consisted of 41 missense mutations, 10 insertion/deletions, 12 synonymous mutations and 31 somatic homozygotes indicating a loss of heterozygosity (LOH). The nonsynonymous variants were assessed for deleterious effect using the consensus derived from three prediction algorithms: Provean (12), SIFT (13) and PolyPhen2 (25) (Table 4).

**Table 4.** Somatic coding mutations identified among 313 cancer genes from a colorectal cancer

Gene	Chr	Position	Mutation	Nr of reference reads, Nr of variant reads, Ref, Var (%)	Amino acid alteration
*EPHA8*	1	22,903,313	c.763G>A	10, 8, (44.44%)	p.V255M
*BCL11A*	2	60,688,795	c.1252A>G	22, 8, (26.67%)	p.S418G
*FGFR3*	4	1,808,557	c.2176T>C	23, 18, (11%)	p.Y726H
*TRIO*	5	14,394,211	c.4283G>A	129, 68, (34.52%)	p.R1428Q
*BRAF*	7	140,453,136	c.1799T>A	145, 46, (24.08%)	p.V600E
*MLL3*	7	151,945,007	c.2512G>A	13, 15, (53.57%)	p.G838S
*PTCH1*	9	98,240,362	c.1322G>A	95, 29, (23.39%)	p.R441H
*HRAS*	11	534,304	c.19G>C	32, 24, (42.86%)	p.V7L
*PML*	15	74,315,480	c.914A>G	24, 7, (22.58%)	p.Y305C
*CREBBP*	16	3,788,671	c.4283G>A	84, 43, (33.86%)	p.R1458H
*MAP2K2*	19	4,101,030	c.692G>A	9, 8, (47.06%)	p.R231H
*NOTCH3*	19	15,292,574	c.2605G>T	31, 10, (24.39%)	p.G869C
*DNMT3B*	20	31,380,486	c.976G>A	58, 46, (44.23%)	p.E326K
*ACVR2A*	2	148,683,686	c.1303delA	47, 39, (45.35%)	-
*TGFBR2*	3	30,691,872	c.449delA	152, 114, (42.86%)	-
*PIK3CB*	3	138,413,710	c.346delC	222, 117, (34.51%)	-
*MECOM*	3	168,833,257	c.1839delA	180, 37, (17.05%)	-
*FLT4*	5	180,055,897	c.1088delC	32, 12, (27.27%)	-
*CCND3*	6	41,903,745	c.224_225insC	101, 34, (25.19%)	-
*NF1*	17	29,553,477	c.2026_2027insC	415, 86, (17.17%)	-
*TCF4*	18	52,895,520	c.2258delC	117, 66, (36.07%)	-
*PLCG1*	20	39,798,133	c.2738_2739insG	70, 16, (18.6%)	-

Reference sequence reads (Ref).

Variant-containing reads (Var).

Var% = variant reads over total.

Number (Nr).

Notably, the colorectal cancer had a *BRAF* V600E somatic mutation. This particular mutation leads to oncogenic activation of the RAS/RAF pathway and is seen in ∼10% of colorectal cancers (26). This mutation is frequently identified in colorectal tumors having microsatellite instability (MSI) (19), a molecular phenotype related to loss of DNA mismatch repair and hypermutability (27). Confirming that this individual had Lynch syndrome, we identified a germline mutation in DNA mismatch repair genes (*MLH1*). It is considered to be clinically actionable with a number of target therapies (i.e. PLX4032) inhibiting its oncogenic activity in melanoma. In contrast, colorectal cancers with this mutation do not respond to therapies targeting this specific *BRAF* mutation as a result of feedback activation of the epidermal growth factor receptor (EGFR) (26).

A series of other coding mutations pointed to this colorectal tumor having MSI in which a tumor rapidly accumulates small insertion and deletion mutations in sequence tandem repeats (28). For example, we discovered a mutation in the*TGFBR2* gene involving a deletion in the homopolymer (A)_10_ tract in exon 4 (28). *TGFBR2* encodes a receptor for the TGF-β pathway and is a known cancer driver gene. This specific coding region microsatellite is a known mutation hotspot in MSI-positive colorectal cancer (CRC) and markedly reduces mRNA levels, presumably due to nonsense-mediated decay (29). Another microsatellite deletion was detected in the homopolymer (A)_14_ sequence and is proximal to exon 3 of the gene *FBXW7* (30). This gene is an ubiquitin protein ligase and facilitates the proteasomal degradation of target proteins involved in cancer such as CCNE (e.g. cyclin-E) and MYC (e.g c-MYC). We identified a deletion leading to a frameshift at codon 435 in the tumor suppressor gene *ACVR2A*. This gene is often mutated in MSI positive CRC (58.1%) (31). This colon cancer also had a R1458H mutation in the HAT domain of *CREBBP*. This gene encodes for a histone acetyltransferase and transcriptional co-activator in multiple signaling and developmental pathways. Ionov *et al.* reported that *CREBBP* is mutated in 85% of the MSI-positive CRC cell lines they tested. This gene regulates transcription of the *TP53* tumor suppressor via histone acetylation (32).

### Application in contiguous genomic loci sequencing

Assay 4 targets a 1.5 Mb region at chromosomal locus 18q21.1 (9213 primer probes) and a 0.2 Mb region at chromosomal locus 3q25 (2517 primer probes). The 18q21.1 locus has been implicated in increasing susceptibility to colorectal cancer (33,34). The *TIPARP* gene is located at 3q25 and is a poly ADP-ribose polymerase. Recent genome wide association studies (GWASs) have shown that *TIPARP* is a susceptibility locus for ovarian cancer, namely, an intergenic SNP was highly significantly associated (*P* = 1.5 × 10–28) with ovarian cancer risk (35) although the potentially causal variants remain unknown. Assay 4 also includes 3334 primer probes selecting 1701 SNPs within 1 Mb of the *TIPARP* locus and the exons of 46 genes associated with breast cancer.

To test this assay, we analyzed normal diploid DNA samples from four different individuals. We sequenced two samples per lane with indexing. With a single GAIIx lane, the average coverage for the 1.5 Mb chromosome 18q21.1 locus over the four samples was 68× and average percentage of detected SNPs in this region present in dbSNP137 was 98.6%. For the 3q25 *TIPARP* locus, a higher density of primer probes was used to cover a 0.2 Mb interval. This produced an average coverage of 163× for the four samples. For the variants called from the *TIPARP* locus, 96.5% SNVs were annotated in dbSNP. There was minimal experimental variance between the samples in regard to sequence yield and coverage (Table 5). As another assessment of variant calling accuracy, we compared the SNPs targeted to genotyping data from an Illumina Infinium HD BeadChip (Supplementary Tables S15, S16, S17 and S18). The concordance between the SNPs detected with Illumina BeadChip and the targeted sequencing was higher than 97% for all of the samples (Table 5).

**Table 5.** Contiguous locus sequencing

Locus	Sample	5614	6253	5613	5326
	Average coverage	152	171	163	168
*TIPARP* locus (200kb) Chr 3: 156299721–156500330	SNPs called	372	294	314	312
	Percentage in dbSNP	363 (97.6%)	286 (97.3%)	298 (94.9%)	300 (96.2%)
	Average coverage	63	72	68	70
18q21.1 locus (1.5Mb) Chr 18: 47749745–49250310	SNPs called	1690	1673	1798	1747
	Percentage in dbSNP	1667 (98.6%)	1653 (98.8%)	1771 (98.5%)	1720 (98.5%)
	Average coverage	74	84	82	80
	SNPs called	2497	2515	2484	2566
Assay 4	Percentage in dbSNP	2467 (98.8%)	2486 (98.8%)	2447 (98.5%)	2528 (98.5%)
	SNV concordance to control BeadChip array	98.0%	99.2%	97.9%	98.4%

The coverage across the two loci was relatively even. However, there were a number of genomic intervals of lower coverage. On further examination of the 1.5 Mb targeted interval, there were 18 regions greater than 0.5 kb that had no sequencing coverage. These regions were the same between the different samples and thus attributable to failures in individual primer probes or probes in highly repetitive regions. These gaps in coverage can be rectified by designing additional primer probes for these regions and spiking them into the original pool.

### Detection and resolving rearrangement breakpoint sequences

**Figure 4.**
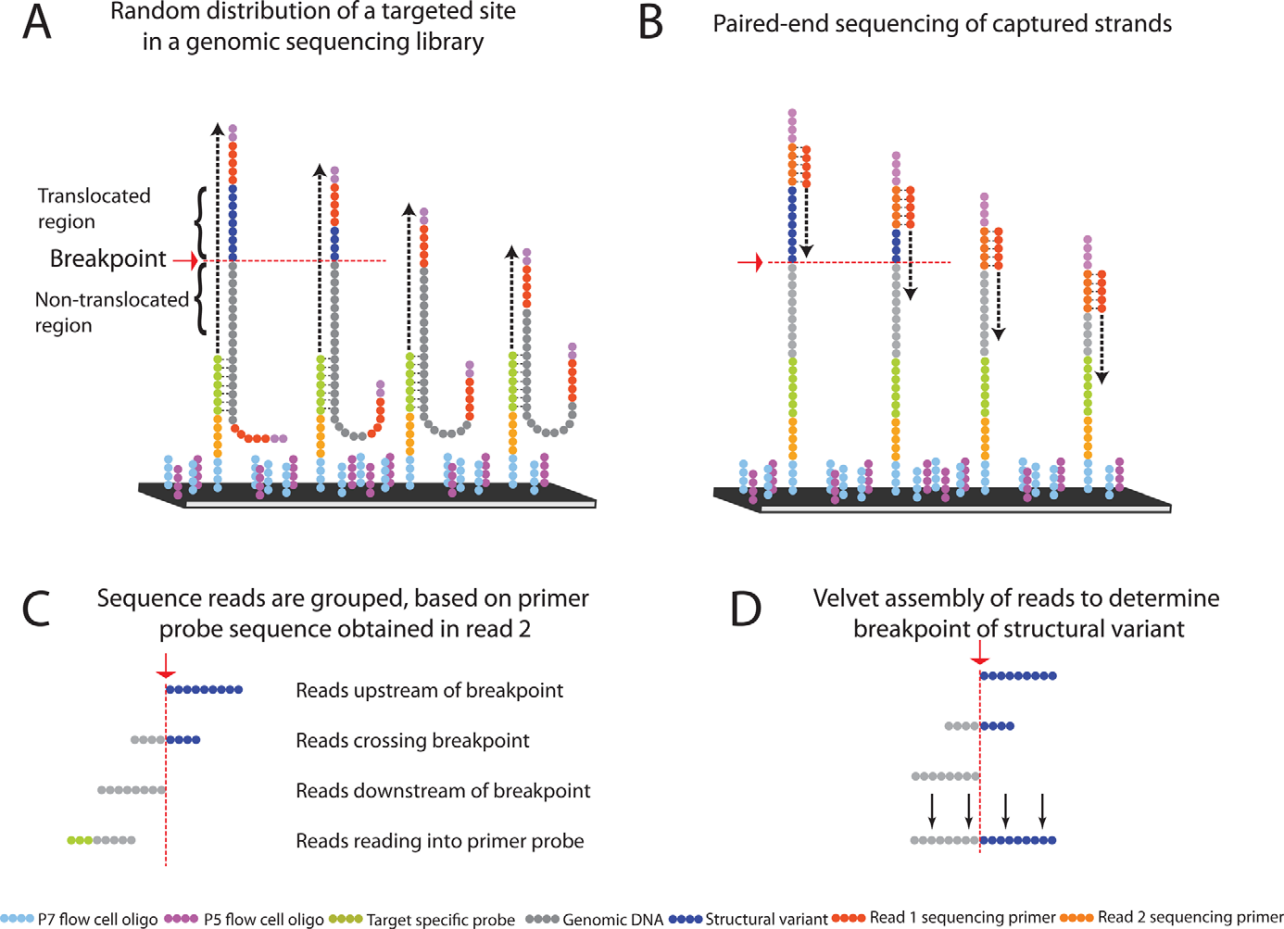
Primer-probe-based determination of rearrangement breakpoints. Exact breakpoint determination by Velvet assembly of reads belonging to a specific primer probe. As a result of random fragmentation of genomic DNA, targeted sites are randomly distributed in the sequencing library (**A**). Following capture of and sequencing of the targeted breakpoint regions (**B**), reads are grouped based on the primer probe sequence obtained in Read 2. Non-aligning reads were extracted to purify reads containing the breakpoint, which is not expected to align as a result of multiple genomic regions in a single read. This target-specific selection of input data circumvents issues of traditional assembly such as limited input data and overrepresentation of wild-type allele. Remaining reads are upstream, crossing or downstream of the structural variant breakpoint, or read into the primer probe oligonucleotide sequence and are discarded (**C**). A new contig is assembled with the remaining reads using the assembly program Velvet (**D**).

To analyze structural variations such as large deletions, insertions and rearrangements, we utilized the synthetic primer probe sequence occurring in Read 2 and used this genomic coordinate information to inform the analysis of the target genomic region in Read 1 (Figure 4). To this end, we designed Assay 5 to target putative rearrangements identified from the whole genome sequencing of two matched primary and metastatic tumor sites (designated as Tumor 1 and 2, respectively) from the same individual and determine the precise breakpoint sequences. We also sequenced a matched normal sample. The Tumor 1 and Tumor 2 samples had a tumor cellularity of ∼40% and 60%, respectively, thus making rearrangement calling more problematic from lower sequencing coverage analysis. From the whole genome sequence, the data were aligned with BWA with an average genome wide coverage of 80× for Tumor 1, 30× for Tumor 2 and 50× for the normal tissue.

Using the program Breakdancer (36), we identified rearrangement candidates within or near exons. These candidates were not found in the normal diploid genome sequence. The criteria for calling a structural variant relied on a minimum of two algorithms calling a breakpoint with at least 50% overlap of the genomic coordinates. Using this method, 1239 tumor-specific candidates were identified including 1011 deletions, 205 insertions and 23 inversions. Among these putative variants, we chose 43 of these events that overlapped exons and 23 extra-genic events in the proximity of known cancer genes. Three control structural variants included one inversion, one deletion and one insertion. They were chosen from a list of germline variants found in the normal diploid DNA sample, and their presence was confirmed in the matched tumor samples.

For this assay, we designed four primer probe sequences flanking each putative breakpoint associated with a structural variant candidate (Figure 4). This assay covered a total of 66 putative breakpoints. The primer probe sequences were selected on the opposing forward and reverse strands surrounding a target putative breakpoint. Primer probes were within a range of 150–300 bases from the target variant breakpoint. As part of the design process, we eliminated any sequences that fell within repetitive regions. In this particular experiment, we generated 80 by 40 paired-end reads from the tumor and normal samples, multiplexed on a single lane. As a result of random fragmentation of genomic DNA in the library preparation, breakpoints of structural variants will be randomly distributed within the library (Figure 4A) and thus within the sequence reads (Figure 4B).

From Read 2, we used the primer probe sequence to group the sequence data for each rearrangement breakpoint candidate. Generally, each rearrangement locus group was covered by more than several hundred reads based on the combination of four primer probes. Using the aggregated sequence data organized by rearrangement locus group according to a specific candidate arrangement candidate, we analyzed the genomic target sequence from Read 1.

For any given individual primer probe, the sequences from a putative structural variant breakpoint can be classified into four categories (Figure 4C): (i) reads overlapping primer probes and reading into its oligonucleotide sequence; (ii) reads that are downstream of the structural variant and do not contain the structural variant sequence; (iii) reads that cross the structural variant breakpoint; (iv) reads that are upstream of the structural variant. Using BWA set at default settings, we identified all the Read 1 sequences that fully aligned to the reference genome (e.g. hg19); these fully aligning reads did not cross over the rearrangement breakpoint and thus were not useful for assembling the breakpoint sequence.

Per each rearrangement locus group, a subset of the reads remained that did not fully align to the reference genome (Table 6). A proportion of these non-aligning reads were likely to incorporate the breakpoint junction and thus create a novel sequence. For each candidate rearrangement breakpoint data set, we conducted local assembly on the remaining non-aligning Read 1 sequences (Figure 4D). For this local process, we used the assembly program Velvet (15). Afterward, the assembled local contigs were aligned with megablast and filtered based on location on the correct chromosome, discontinuous sequence alignment starting from the breakpoint and appearance only in the cancer genome. We removed megablast alignment results that were assigned to the wrong chromosome and structural variants that were also present in the matched normal tissue. The germline variants used as a control were confirmed. We validated a total of eight somatic rearrangements as noted in Table 6. These were not found in the matched normal DNA sequence. The other rearrangement candidates had no evidence of somatic breakpoint sequences.

**Table 6.** Validated cancer rearrangements

	Nr of non-aligning sequence reads per locus			Assembled contig(s) with a novel breakpoint sequence					
Candidate somatic structural variant	Tumor 1	Tumor 2	Normal tissue	Tumor 1	Tumor 2	Normal tissue	Chr	Validated position of breakpoint 1	Validated position of breakpoint 2
Deletion	499	396	644	Yes	Yes	-	7	38386883	38397600
Inversion	629	313	613	Yes	-	-	15	99301834	101056862
Deletion	348	190	339	Yes	Yes	-	15	100686976	100693180
Inversion	220	120	238	Yes	-	-	16	55794659	55867118
Deletion	186	96	208	Yes	Yes	-	16	68847304	68847405
Deletion	188	48	151	Yes	Yes	-	19	6493052	6498221
Deletion	397	218	451	Yes	Yes	-	19	21268385	21334585
Deletion	175	82	223	Yes	-	-	22	23959245	23965974

Number (Nr).

## DISCUSSION

We developed OS-Seq, an approach for targeted detection of genomic variants, such as cancer mutations and structural variants. As we demonstrate, OS-Seq allows one to program a sequencer for targeting genomic regions. Compared to our first effort, we substantially improved the workflow automation; target enrichment and flow cell preparation for sequencing entirely take place on a standard fluidics device. The actual experimental manipulation is limited to easily automatable library preparation without the need for size selection, minimizing experimental hands-on time.

Since target selection is integrated with flow cell preparation for sequencing, instead of a part of the library preparation step, increasing assay target size is a simple matter of combining primer probe pools prior to the flow cell modification step. As new candidate regions or genes are identified and require follow-up sequencing, oligonucleotides from either column or array-synthesized sources can easily be added to increase the feature size and applications for any given targeting assay.

We also refined the primer probe design and this resulted in a large increase in the percentage of primer probes yielding on-target sequencing. OS-Seq's library target-extension approach also ensures high on-target sequence yield; for a sequencing cluster to form an individual sequencing library, DNA strand must hybridize to a flow cell bound primer probe and undergo a polymerase extension reaction. However, we still observe a proportion of reads that are off-target. As described in variant calling comparisons, the majority of these off-target reads are derived from homologous genes with shared sequence motifs. When using sequence-based enrichment strategies, there will always have to be a consideration of the specificity of the probes versus the number of probes in a certain targeted region. The overall uniformity of targeting also improved as a result of the refined primer probe design. For future studies, we will attempt to alter the concentration of individual primer probes, which expectedly would improve uniformity even further.

Many clinical samples are complex mixtures where there are multiple, distinct genetic contributors (e.g. infiltrating normal tissue or clonal subpopulations in a tumor). In addition, each source's contribution may vary and this leads to quantitative differences as seen in MAF (6). With very high number of reads originating from targeted regions, deep sequencing has higher sensitivity to detects less prevalent, minor alleles and mutations from such admixed samples. We demonstrated the application for detecting mutations from cancer genomes and low minor allele frequencies proportionally.

There are several ways of improving the targeting performance. Foremost, we can increase the sequencing depth by reducing the amount of targets per lane or by increasing the total yield. Currently, we obtain cluster yields close to 70% of standard Illumina sequencing capacity with the vast majority of which are on-target sequence. We have indications that the cluster reaction is less efficient compared to standard flow cell preparation, resulting in loss of some clusters due to low intensity. However, as a result of OS-Seq's library capture-extension approach, every captured library strand does give rise to a single cluster and thus a sequencing read, so there is no sequence capacity loss due to post-capture PCR duplication. Yield is not sensitive to small changes in sequencing library concentration, so exact titration of the sequencing library is not required.

When analyzing variants in targeted regions (e.g. *FANCD2*), we observed that variant containing sequence reads were sometimes filtered as a result of low mapping quality when using single individual reads from mate pairs. We used this individual read alignment method to minimize misalignment as a result of the synthetic primer probe sequence occurring in Read 2. Lower mapping qualities may be improved if we use paired-end reads for alignment and eliminate the synthetic sequence. We are working to optimize paired-end alignment. In addition, single-molecule tagging methods or use of statistical variant-calling algorithms (6) are other possibilities to further improve detection capability.

With the completion of many GWASs and identification of specific loci associated with disease phenotype, there is increasing interest in identifying the causal rare variants that are in linkage disequilibrium with identified SNPs (37). We demonstrated the high concordance to an Illumina BeadChip array of an assay that, next to 29 cancer related genes, covers a contiguous locus of 1.5 Mb while simultaneously genotyping 1701 specific candidate SNPs of interest from GWAS studies. To cover sequencing gaps in the contiguous interval, one solution will be to design new primer probes targeting the gap regions.

Determining the genomic sequence of structural variation such as rearrangements, large insertions, inversion, deletions, etc., is a nontrivial task. Frequently, it requires extensive computational analysis, typically on whole genome sequencing data, followed by independent experimental validation for confirmation of the rearrangement breakpoint sequence. The difficulty of validating rearrangement breakpoints increases when one is dealing with heterozygous structural variations or lower allelic frequencies as a result of a genetic mixture, such as the tumor sample that was used for validation of mutations and putative rearrangement breakpoints in this study. We applied targeted sequencing for breakpoint validation. As we demonstrated (4), the majority of target reads align up to 400 bases from the primer probe. Therefore, one must design primer probes within several hundred bases of a putative rearrangement breakpoint. In the case of our tumor samples, the majority of reads will not contain the structural variant breakpoint given the high proportion of normal genome. We did confirm structural variants at exact breakpoint sequence resolution, although a proportion of putative breakpoints were not validated either as a result of lack of adequate coverage or potential false positives arising from the initial structural variant calling. By further increasing the coverage, we should be able to improve the breakpoint identification.

We are continuing to make improvements to OS-Seq, which will include the addition of single-molecule detection with barcoding and testing this technology on sequencers that have turnaround of two days. We anticipate that these future developments will facilitate the potential adoption of this technology into a clinical setting.

## AVAILABILITY

The primer probe design pipeline is available for public download on http://dna-discovery.stanford.edu/software/osseq/.

## SUPPLEMENTARY DATA

Supplementary Data are available at NAR Online.

SUPPLEMENTARY DATA
