# Supplementary material for: A programmable method for massively parallel targeted sequencing
Source: Nucleic Acids Res. 2014 Apr 29;42(10):e88. doi: 10.1093/nar/gku282 (PMC4041455; doi:10.1093/nar/gku282)
Supplement: SUPPLEMENTARY DATA [file supp_42_10_e88__index.html]

A programmable method for massively parallel targeted sequencing — A programmable method for massively parallel targeted sequencing — SUPPLEMENTARY DATA 

# A programmable method for massively parallel targeted sequencing

## SUPPLEMENTARY DATA

**Files in this Data Supplement:**

- SUPPLEMENTARY DATA
- SUPPLEMENTARY DATA
- SUPPLEMENTARY DATA
- SUPPLEMENTARY DATA
