# Supplementary figures and images for: A programmable method for massively parallel targeted sequencing
Source: Nucleic Acids Res. 2014 Apr 29;42(10):e88. doi: 10.1093/nar/gku282 (PMC4041455; doi:10.1093/nar/gku282)

Supplementary Figure S1

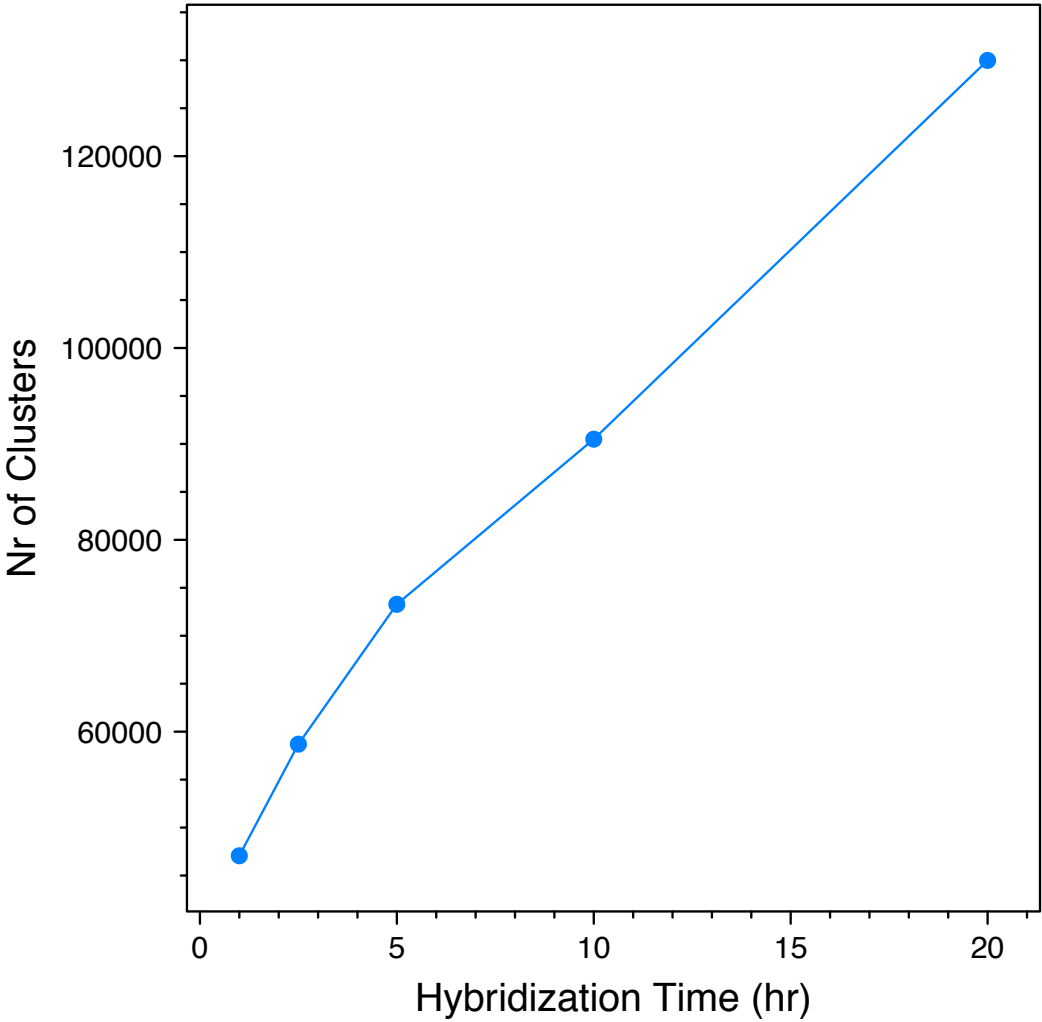

Supplementary Figure S2

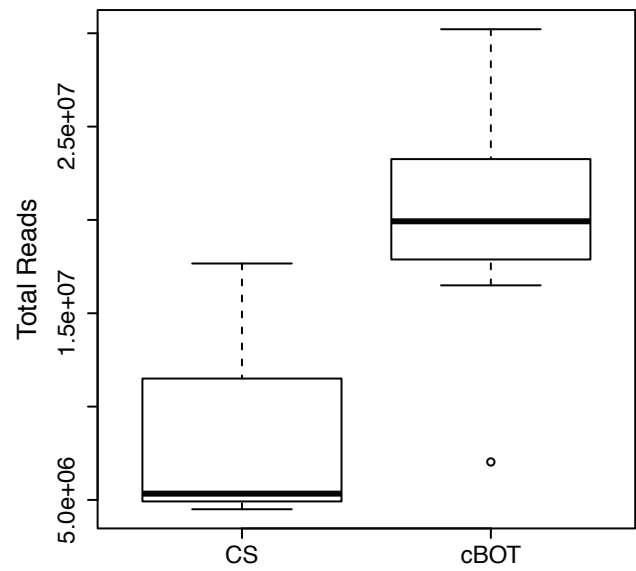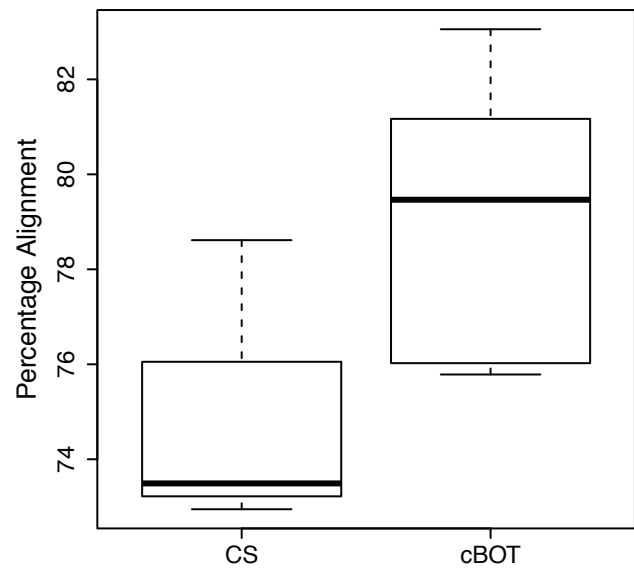

Supplement: SUPPLEMENTARY DATA [file supp_gku282_nar-00250-met-k-2014-File006.pdf]
